# Exposure Keratopathy: An Idiopathic Lagophthalmos Case Report

**DOI:** 10.7759/cureus.18945

**Published:** 2021-10-21

**Authors:** Khalid Alhoutan, Khalid Alarfaj

**Affiliations:** 1 Ophthalmology, Ad Diriyah Hospital, Ad Diriyah, SAU; 2 Ophthalmology, King Fahd University Hospital, Dammam, SAU

**Keywords:** anterior segment, lagophthalmos, keratitis, exposure keratopathy, cornea

## Abstract

Exposure keratopathy refers to corneal damage that results primarily from prolonged exposure of the ocular surface to the outside environment. Herein, we describe a case of exposure keratopathy with bilateral idiopathic lagophthalmos and discuss factors pertaining to prompt diagnosis and treatment.

A 21-year-old woman presented with bilateral nocturnal lagophthalmos, blurred vision, and whitish spots in both eyes. She had no remarkable history of medication use, trauma, surgery, cranial nerve abnormality, critical illness, or other ocular problems. Examination revealed bilateral lagophthalmos, good Bell’s phenomenon, bilateral inferior corneal scars, and vision loss. Laboratory results were normal; there was an absence of proptosis, and no epithelial defects were apparent. Based on these findings, she was diagnosed with exposure keratopathy resulting from idiopathic bilateral lagophthalmos and treated with lubricants.

This was a rare case of exposure keratopathy with bilateral lagophthalmos of idiopathic origin, and a challenging one, which prompted the researchers to formulate an appropriate treatment plan.

## Introduction

Exposure keratopathy is characterized by dryness of the cornea with subsequent epithelial breakdown. It may be due to inadequate eyelid closure, which results in reduced lubrication of the ocular surface by tears [1,2]. Eyelid closure and blinking are responsible for replenishing and spreading the tear film across the corneal surface and restricting evaporation of the tear film [3]. Unclosed eyelids and incomplete blinking are the main causes of exposure keratopathy [4]. Incomplete eyelid closure or lagophthalmos is a major risk factor for exposure keratopathy [5,6]. Exposure keratopathy reportedly develops in 3.6-60% of patients who stay in ICUs [5,6].

A wide range of pathologies can result from prolonged exposure of the corneal surface. One is infectious keratitis, and the associated disintegration of the epithelium and loss of antimicrobial components of the tear film such as beta lysin, lactoferrin, and immunoglobulins can increase susceptibility to infection. Another potentially devastating presentation is corneal thinning, which can progress to full-thickness perforation [7,8]. In addition, slit lamp examination can reveal various manifestations including punctuate and macro epithelial erosion, stromal opaqueness (whitening) with epithelial defects, and stromal scars. All of these manifestations tend to occur more commonly in the lower part of the cornea [9].

Clinical presentations of exposure keratopathy include blurred vision, eye irritation, red eyes, and dry eyes. In severe cases, the condition can lead to corneal ulcers and microbial keratitis [10]. Common signs include incomplete blinking, lagophthalmos, reduced tear meniscus, reduced tear film breakup time, corneal filament formation, punctate epithelial erosion, and epithelial defects [11,12].

## Case presentation

A 21-year-old Saudi woman presented to the Anterior Segment Clinic at King Fahad University Hospital in Al-Khobar City, complaining of whitish spots in both eyes, which had developed over the past three years. The spots had gradually increased in size, and there was an associated blurring of vision that could not be corrected via refraction. She had experienced intermittent nocturnal lagophthalmos for years before the present complaint was noted. She was not taking any systemic or ocular medication. There was no family history of lagophthalmos or any ocular problems, and no history of trauma, surgery, redness, or discharge.

**Figure 1:**
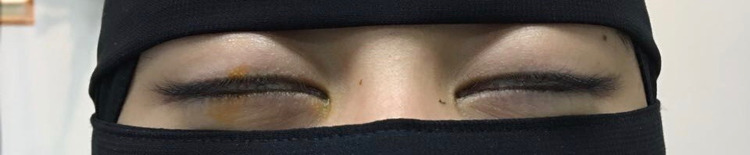
Bilateral lagophthalmos measuring 2 mm in width.

Visual acuity without correction showed 0.5 loss in the right eye and 0.6 in the left, with no improvement by refraction. The pupils were round, regular, and reactive in both eyes without relative afferent defect. Intraocular pressure was 15 mmHg in both eyes. External examination of the ocular apparatus revealed 2-mm wide lagophthalmos in both eyes and a clear Bell’s phenomenon (Figure 1). Proptosis was ruled out via Hertel exophthalmometry, which yielded measurements of 16.1 mm bilaterally. Extraocular muscle movements were normal and exhibited the full range of motion. Examination of cranial nerves III, IV, V, VI, and VII shows them to be intact and fully functional.

**Figure 2:**
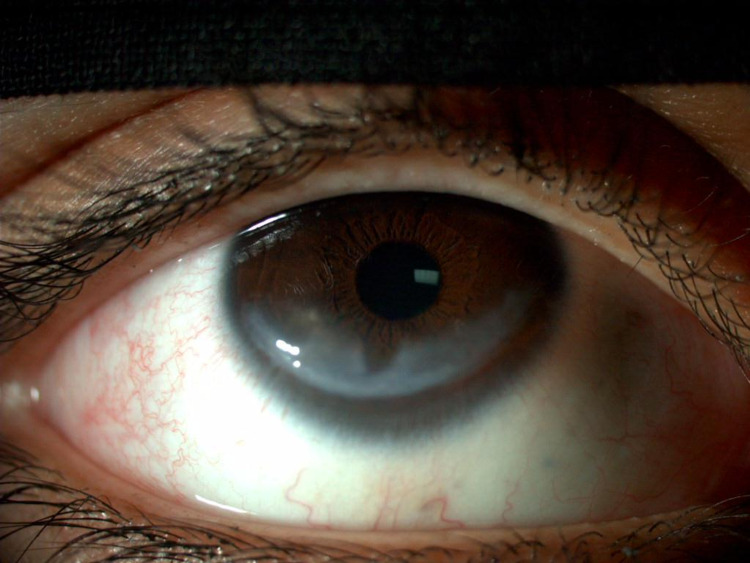
Right eye with an inferior corneal white scar measuring approximately 9 x 4 mm.

**Figure 3:**
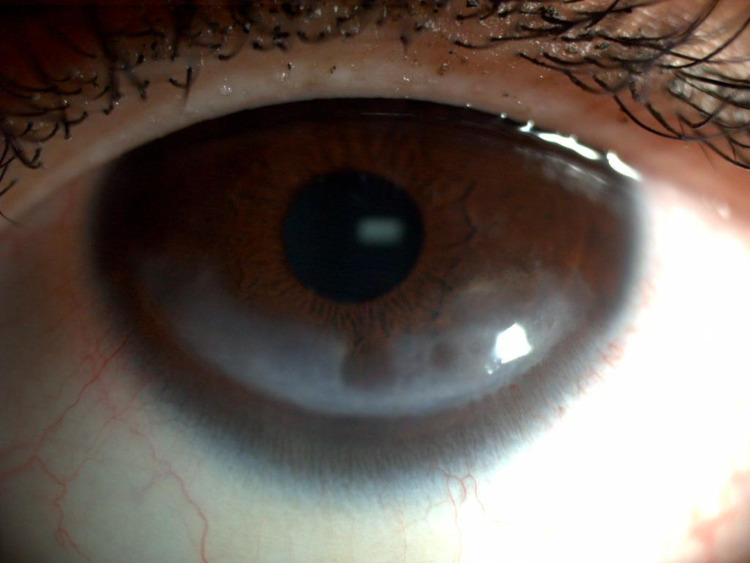
Left eye with an inferior corneal white scar measuring approximately 9 x 4 mm.

Slit lamp examination revealed mild blepharitis in both eyes and conjunctiva/sclera were quiet in both eyes. Bilateral 9 x 4-mm inferior corneal scars with a central area of stromal thinning were observed in both eyes (Figure 2 and Figure 3). No epithelial defect or corneal infiltrate was evident. Both anterior chambers were deep and quiet. Both irises exhibited normal architecture, and both lenses were clear with normal fundi. Laboratory investigations including complete blood count, liver function tests, renal function tests, thyroid function tests, fasting blood glucose, and lipid profile yielded results that were all within normal ranges. Based on these findings, the patient was diagnosed with bilateral exposure keratopathy. She was prescribed a lubricating solution to replenish the tear film and advised to apply it every four hours while awake, and before going to sleep each night.

## Discussion

The patient underwent a thorough and complete examination to investigate possible etiologies for exposure keratopathy. She presented with bilateral lagophthalmos, which is a major risk factor for exposure keratopathy. The incidence of exposure keratopathy is relatively high in ICU patients, but the patient had not stayed in an ICU. 

The etiologies of lagophthalmos can be broadly classified into three categories [13]. The first category is proptosis-related causes such as physiological abnormalities, intraorbital tumors, or endocrine-related conditions including dysthyroid optic neuropathy/thyroid eye disease. It is important to investigate potential thyroid eye disease, especially in female patients. Notably, the patient yielded normal exophthalmometry and laboratory results; thus these potential causes were excluded. Many systemic diseases, especially neurological conditions, have also been implicated as causative or linked to exposure keratopathy. Facial nerve paralysis has been known to cause lagophthalmos with exposure keratopathy as a subsequent consequence. Because the facial nerve innervates the facial muscles, it affects facial symmetry and eyelid closure and is integral in the prevention of paralytic lagophthalmos [14]. Another important cranial nerve is the trigeminal nerve, which supplies sensory input to the cornea. Trigeminal nerve lesions can lead to neurotrophic keratitis that inhibits the blinking reflex, which is vital for protecting the cornea from external stimuli and normal tear film production [15]. However, the patient had no history or signs of neurological conditions. Furthermore, no potentially contributary conditions such as sleep apnea with consequent sequelae [16], or floppy eyelid syndrome were reported by the patient.

The second category of lagophthalmos etiologies is palpebral pathology-related causes. Abnormal eyelid closure can be caused by previous surgery or a traumatic injury, neither of which were applicable in the current case.

The third category of lagophthalmos etiologies is idiopathic origin. Because the current patient had no history of known risk factors for lagophthalmos, we diagnosed her with idiopathic lagophthalmos. A simple lubricant applied to both eye surfaces (Lacri-lube) was prescribed as treatment because the case was deemed mild and lubricant treatment reportedly does not differ significantly from polyacrylamide hydrogel dressings [17]. It has been suggested that prosthetic replacement of the ocular surface ecosystem (PROSE) is a good treatment option, but to date, there is a lack of available studies supporting PROSE. The primary aim is the improvement of the patient’s vision [4,18], which is now fully functional after treatment. Tantalum, gold, and platinum upper eyelid weights remain under consideration, depending on the progression of the case. Other invasive treatment options include permanent tarsorrhaphy, palpebral springs, and manipulation of the upper lids via levator muscle and Müller’s muscle recession.

Identifying the underlying cause of the condition remains a priority, and doing so would facilitate the more informed development of a treatment plan. The case remains challenging due to the idiopathic nature of the lagophthalmos and emphasizes the need for appropriate management guidelines for exposure keratopathy as a consequence of idiopathic lagophthalmos.

## Conclusions

Exposure keratopathy with bilateral lagophthalmos of idiopathic origin is rare, and it is challenging with regard to prompt diagnosis and treatment. The young woman in the present case was diagnosed by exclusion as all other etiologies were thoroughly investigated. We hope this case may lead researchers to discuss more appropriate management plans to help prevent idiopathic lagophthalmos, which results in exposure keratopathy, at an early stage and to find proper treatment plans.
